# P-1354. Outcomes of Patients Treated With Cefiderocol For Infections Caused by β-Lactam–β-Lactamase Inhibitor Non-Susceptible Bacteria: Subgroup Analysis of the PROVE Study

**DOI:** 10.1093/ofid/ofaf695.1542

**Published:** 2026-01-11

**Authors:** Ryan K Shields, Mathias W Pletz, Maria Cruz Soriano Cuesta, Stefano Verardi, Karan Gill, Anne Santerre Henriksen, Sean T Nguyen

**Affiliations:** University of Pittsburgh, Pittsburgh, PA; Institute of Infectious Diseases and Infection Control, Jena University Hospital/Friedrich-Schiller-University, Jena, Germany, Jena, Thuringen, Germany; Ramón y Cajal University Hospital, Madrid, Spain, Madrid, Madrid, Spain; Shionogi, B.V., London, England, United Kingdom; Shionogi, London, England, United Kingdom; Shionogi BV, London, UK, London, England, United Kingdom; Shionogi Inc., Florham Park, NJ

## Abstract

**Background:**

*In vitro* datiderocol and newly developed β-lactam–β-lactamase inhibitors (BL–BLIs). The PROVE study enrolled patients with serious Gram-negative bacterial infections treated with cefiderocol. We compared patient characteristics, pathogens, and clinical outcomes by susceptibility status to BL–BLIs.

**Figure:**
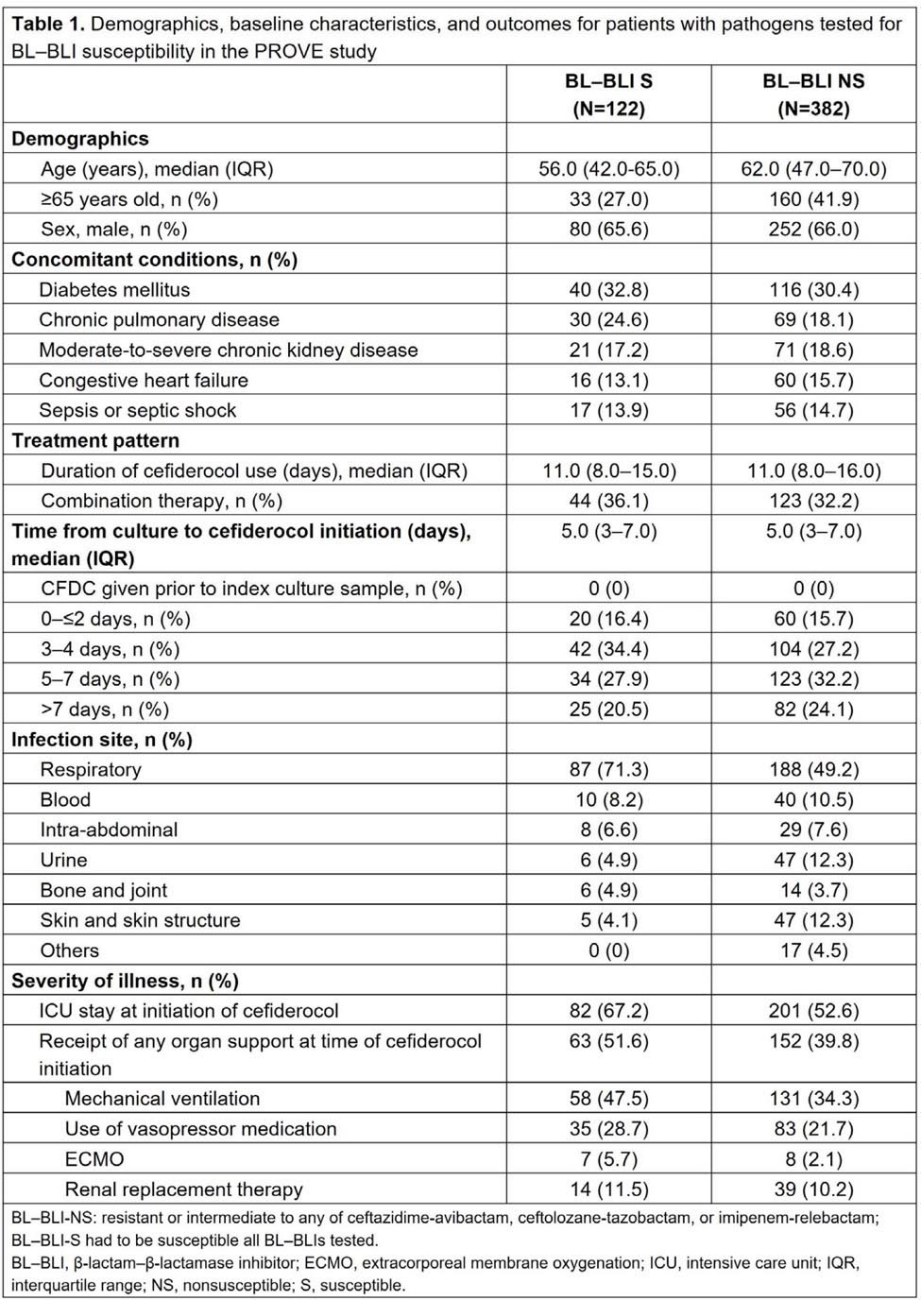

**Figure:**
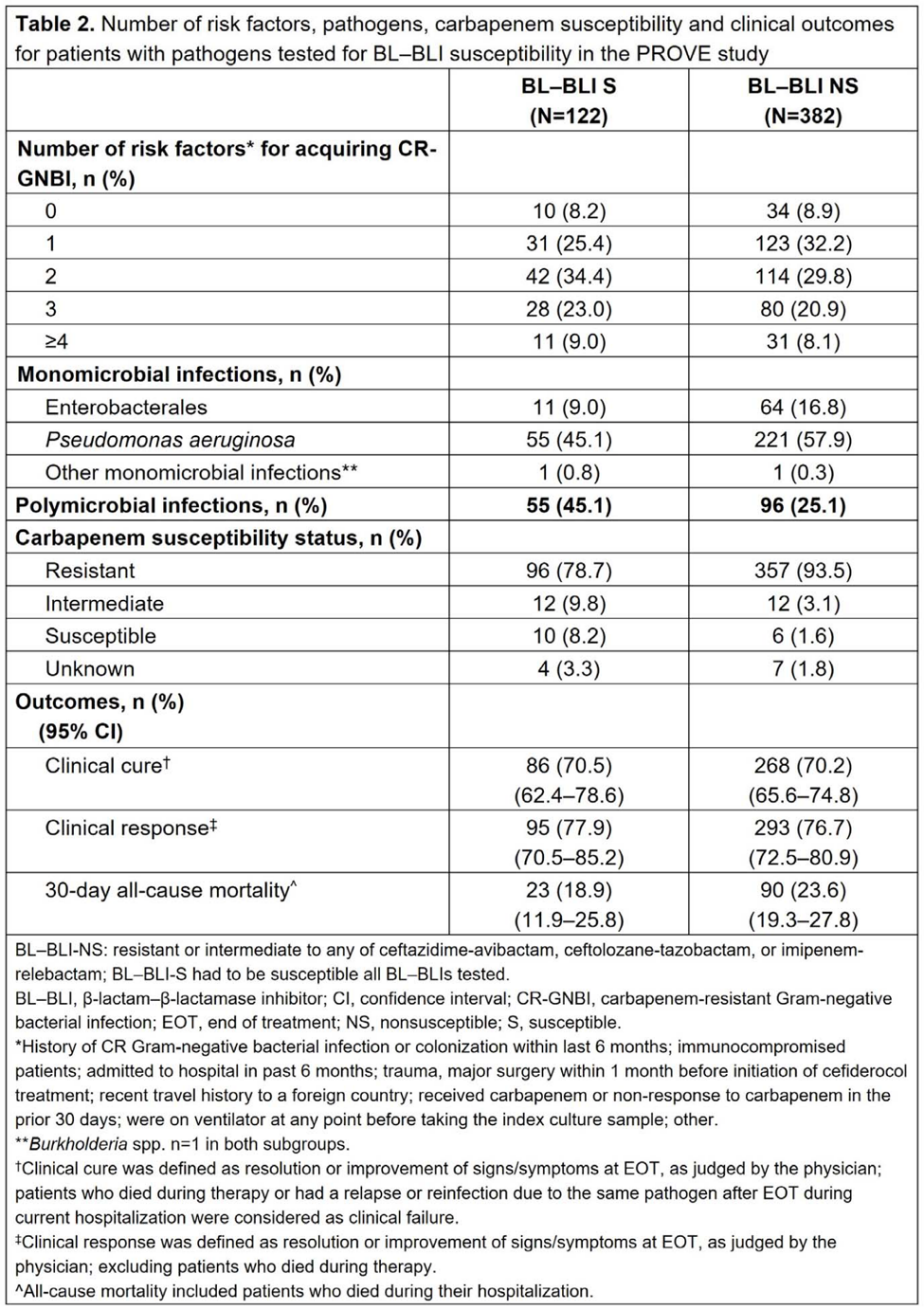

**Methods:**

PROVE was an observational medical chart review study (November 2020–July 2024). Data from hospitalized patients with confirmed Gram-negative bacterial infections and known BL–BLI susceptibility who received cefiderocol for ≥72 hours were included. Susceptible bacteria were susceptible to all BL–BLIs tested (S); non-susceptible bacteria were resistant or intermediate to at least one BL–BLIs tested (NS): ceftazidime-avibactam, ceftolozane-tazobactam, and imipenem-relebactam. Baseline demographics, clinical characteristics, and clinical outcomes were assessed.

**Results:**

Among 504 patients, those infected by NS (N=382) vs S (N=122) bacteria were older (median age 62.0 vs 56.0 years, respectively; Table 1). Proportionally, fewer patients with NS vs S bacteria had indicators of more severe disease at cefiderocol initiation (intensive care unit stay: 52.6% vs 67.2%; organ support: 39.8% vs 51.6%). Patients with S vs NS pathogens more frequently had respiratory tract infections (71.3% vs 49.2%; Table 1) and were more likely to have ≥2 risk factors for acquired CR Gram-negative bacteria (66.4% vs 58.9%; Table 2). Polymicrobial infections were more common in patients with S vs NS bacteria (45.1% vs 25.1%, respectively). Nearly all NS bacteria were carbapenem resistant. Clinical cure rates were similar in patients with NS and S bacteria (70.2% vs 70.5%, respectively). 30-day all-cause mortality was numerically lower for patients with S vs NS bacteria (18.9% vs 23.6%, respectively) (Table 2).

**Conclusion:**

Clinical cure rates were similar in patients with BL–BLI-S and NS pathogens, but differences in baseline severity limit comparability. Further analyses are needed to clarify the role of cefiderocol in infections caused by NS pathogens.

**Disclosures:**

Mathias W. Pletz, MD, GSK: Advisor/Consultant|GSK: Honoraria|MSD: Advisor/Consultant|MSD: Honoraria|Pfizer: Advisor/Consultant|Pfizer: Grant/Research Support|Pfizer: Honoraria|Shionogi: Advisor/Consultant|Shionogi: Honoraria Maria Cruz Soriano Cuesta, MD, Gilead: Advisor/Consultant|Gilead: Honoraria|MSD: Advisor/Consultant|MSD: Honoraria|Mundipharma: Advisor/Consultant|Mundipharma: Honoraria|Pfizer: Advisor/Consultant|Pfizer: Honoraria|Shionogi: Advisor/Consultant|Shionogi: Honoraria|Viatris: Advisor/Consultant|Viatris: Honoraria Stefano Verardi, MD, Shionogi BV: Employee Karan Gill, Master of Science, Shionogi BV: Employee Anne Santerre Henriksen, PHD, Shionogi BV: Advisor/Consultant Sean T. Nguyen, PharmD, Shionogi Inc: Employee

